# Improving Detection Accuracy of Lung Cancer Serum Proteomic Profiling via Two-Stage Training Process

**DOI:** 10.1186/1477-5956-9-20

**Published:** 2011-04-17

**Authors:** Pei-Sung Hsu, Yu-Shan Wang, Su-Chen Huang, Yi-Hsien Lin, Chih-Chia Chang, Yuk-Wah Tsang, Jiunn-Song Jiang, Shang-Jyh Kao, Wu-Ching Uen, Kwan-Hwa Chi

**Affiliations:** 1 Division of Chest Medicine, Shin Kong Wu Ho-Su Memorial Hospital, Taipei, Taiwan; 2 Division of Radiation Therapy and Oncology, Shin Kong Wu Ho-Su Memorial Hospital, Taipei, Taiwan; 3 Institute of Traditional Medicine, National Yang-Ming University; 4 Division of Hematology and Oncology, Shin Kong Wu Ho-Su Memorial Hospital, Taipei, Taiwan; 5 School of Medicine and Institute of Biomedical Image and Radiation Science, National Yang Ming University, Taipei, Taiwan

**Keywords:** SELDI, SAA, lung cancer

## Abstract

**Background:**

Surface-Enhanced Laser Desorption/Ionization Time-of-Flight Mass Spectrometry (SELDI-TOF-MS) is a frequently used technique for cancer biomarker research. The specificity of biomarkers detected by SELDI can be influenced by concomitant inflammation. This study aimed to increase detection accuracy using a two-stage analysis process.

**Methods:**

Sera from 118 lung cancer patients, 72 healthy individuals, and 31 patients with inflammatory disease were randomly divided into training and testing groups by 3:2 ratio. In the training group, the traditional method of using SELDI profile analysis to directly distinguish lung cancer patients from sera was used. The two-stage analysis of distinguishing the healthy people and non-healthy patients (1^st^-stage) and then differentiating cancer patients from inflammatory disease patients (2^nd^-stage) to minimize the influence of inflammation was validated in the test group.

**Results:**

In the test group, the one-stage method had 87.2% sensitivity, 37.5% specificity, and 64.4% accuracy. The two-stage method had lower sensitivity (> 70.1%) but statistically higher specificity (80%) and accuracy (74.7%). The predominantly expressed protein peak at 11480 Da was the primary splitter regardless of one- or two-stage analysis. This peak was suspected to be SAA (Serum Amyloid A) due to the similar m/z countered around this area. This hypothesis was further tested using an SAA ELISA assay.

**Conclusions:**

Inflammatory disease can severely interfere with the detection accuracy of SELDI profiles for lung cancer. Using a two-stage training process will improve the specificity and accuracy of detecting lung cancer.

## Background

Lung cancer is the most common malignancy in the world, with poor overall 5-year survival rate[1]. Although advances in non-invasive imaging have improved its detection, > 75% of lung cancer patients present with advanced stages, when therapeutic options are limited[2]. Even patients who present with clinical stage I have at best a 60% 5-year survival rate, signifying that a large percentage of patients regardless of stage have undetectable metastatic disease at the time of presentation [2,3]. Early detection is the most important factor to improve survival. However, the current tools used for early detection are non-satisfactory in accuracy and timing, despite routine chest X-ray, computed tomography, bronchoscopy, sputum cytology, and tumor markers. Current serum biomarkers are convenient but lack of adequate sensitivity and/or specificity [4-8]. Better biomarkers are needed for screening, follow-up, and differential diagnosis.

SELDI-TOF-MS is currently one of the techniques used for cancer biomarker screening, including ovarian, breast, prostate, and liver cancers [9-13]. SELDI profiling reportedly detects lung cancer from healthy individuals [14-20] and most of the SELDI profiling comes from analyzing samples from cancer patients and healthy individuals. However, inflammatory reaction is commonly associated with lung cancer diagnosis. Many identified serum peaks turn out to be the fragments of acute phase proteins. Serum amyloid A (SAA) is a sample of acute phase protein that has been detected by SELDI study. It belonged to a family of apolipoproteins and is reported as a serum biomarker of lung cancer and other malignancies, as well as many inflammatory diseases [14,21-27].

A positive association between previous lung disease, such as chronic bronchitis or pneumonia, with lung cancer has been found [28-30]. Pneumonia frequently co-exists when lung cancer is diagnosed. A study shows that > 50% of newly-proved lung cancer patients have been diagnosed within 3 months after hospitalization for pneumonia[31]. A history of previous lung disease, including asthma, chronic bronchitis, pneumonia, and tuberculosis, is associated with significantly increased risk of lung cancer [28,32].

Thus, differentiating lung cancer from inflammatory disease is an important concern. The impact of inflammatory disease on the detection of lung cancer by SELDI profile has not been highlighted. This study aimed to improve the detection accuracy of lung cancer by SELDI in mixed individuals, those who are healthy and those with inflammatory disease.

## Methods

### Patients and samples

The serum samples from cancer patients were collected from the Department of Chest Medicine of Shin Kong Wu Ho-Su Memorial Hospital. Samples from patients with other diseases and healthy volunteers were collected from the Department of Health Management during this same period. The ethics committee/institutional review board approved the study protocol and all patients provided informed consent.

Each sample was collected into a 10 ml serum separator vacutainer tube and laid up at 4°C for 3 h, and then centrifuged for 10 min at 3000 rpm. The serum was distributed into 100 *μ*l aliquots and stored frozen at -80°C until analysis. A total of 221 serum specimens were collected. In the 118 cancer patients (80 males and 38 females), histologic distribution were 27 squamous cell carcinomas, 71 adenocarcinomas, 9 small cell cancers, and 11 others. Patients with inflammation (23 males and 8 females) included 15 pneumonia, 6 tuberculosis, and 10 urinary tract infection patients. None of them had any evidence of malignancy. The 72 healthy controls (39 males and 33 females) came from general survey of health were used as normal control and had no cancer history or acute/chronic inflammatory disease.

The serum specimens were randomly assigned to a training group and testing group by a 3:2 ratio from the three sets of patients. Thus, the training group had 71 cancer patients (40 adenocarcinoma, 19 squamous cell carcinoma, 5 small cell carcinoma, and 7 other types), 46 healthy controls, and 17 inflammatory disease patients. The testing group has 47 cancer patients (31 adenocarcinoma, 8 squamous cell carcinoma, 4 small cell carcinoma, and 4 other types). The mean age of the training and testing groups was 60 ± 14 and 59 ± 15 years, respectively (*p *< 0.39) and the ratio of male to female was 59%:41% and 67%:33%, respectively (*p *< 0.1), Two groups were matched for sex and age. The demographic information for all collected samples was provided in Table 1.

**Table 1:** Patient characteristics

Histological classification		Training group			Testing group		
		No.		age	No.		age
		M	F		M	F	
Lung cancer	Adenocarcinoma (n = 1)	22		68(52-91)	17		61(32-76)
			18	62(41-86)		14	61(33-76)
	Squamous cell carcinoma (n = 7)	16		68(50-85)	7		65(60-80)
			3	69(56-80)		1	50(50)
	Small cell carcinoma (n = 9)	5		68(64-85)	4		53(48-61)
	Other (n = 11)	5		59(28-74)	4		68(50-81)
			2	53(51-55)			

Non-malignancy	Healthy (n = 72)	23		54(42-67)	16		52(34-81)
			23	51(32-73)		10	46(33-63)
	Inflammatory disease (n = 31)	12		58(36-86)	11		58(27-84)
			5	43(20-74)		3	71(53-92)

### SELDI protein profiling

For analysis, ProteinChip Arrays (CM10) with anionic surface chemistry was used. The CM10 ProteinChip Arrays incorporated a carboxylate group that acted as a weak cation exchanger. The chips were put in a bioprocessor (Ciphergen Biosystems, Inc.), which allowed larger volumes of serum to each chip array. Within the bioprocessor, the chips were equilibrated for 15 min in 150 μl binding/washing buffer (100 mM sodium acetate, pH 4.0) per well. Six-microliter serum samples were denatured in 12 μl urea buffer (9 M urea, 2% CHAPS, and 50 mM Tris). Then, 220 μl of binding buffer were added to the serum mixture, and 100 μl of the denatured, diluted serum were randomly applied to each chip spot. The bioprocessor was then sealed and shaken at a speed of 400 rpm on a platform shaker for 30 min.

After the incubation, the bioprocessor was washed with 150 μl of washing buffer in each well. This step was repeated three times, and each time the binding buffer was discarded by inverting the bioprocessor on a paper towel. The bioprocessor was rinsed with 1 mmol/l HEPES and the chips were removed from the bioprocessor. After the arrays were air-dried, 1 μl of 50% saturated matrix solution (sinapinic acid in 50% acetonitrile and 0.5% trifluoroacetic acid) was applied on the array and allowed to air dry. Arrays were analyzed using a ProteinChip Reader (ProteinChip PBS IIC, Ciphergen Biosystems Inc.), with the following settings: laser intensity 185, detector sensitivity 9, and molecular mass range 2,000-20,000 Da. Analysis were performed in duplicate on serum specimens from each patient in the study group. Mass accuracy was calibrated externally using the all-in-one peptide molecular mass standard (Ciphergen Biosystems, Inc.).

### Peak Detection, Data Analysis, and Decision Tree Classification

Peak detection was performed using Ciphergen ProteinChip Software, version 3.0.2 (Ciphergen Biosystems). The spectra were normalized to total ion current intensity of m/z between 2,000 and 20,000, and baselines were subtracted. The part of the spectrum with m/z values < 2,000 was eliminated for analysis, since the energy absorbing matrix signal usually interfered with peak detection in this range. ProteinChip Software auto-detected the peaks to clusters between 2,000 and 20,000 m/z ratios with a signal-to-noise ratio of > 5. The cluster mass window was 0.3% of mass. The peaks clustered using second-pass peak selection with signal-to-noise ratio > 2. The M/Z values that were within the 0.3% mass accuracy window were regarded as equal between replicates. In order to minimize data variation data between multiple samples, the peak intensity values were logarithmically transformed.

Based on the peak intensities of each signal cluster, three decision trees were constructed from the training groups. For each sample, the intensity values for each peak within the 2,000-20,000 m/z range were inputted into the Biomarker Patterns Software (Ciphergen Biosystems) and classified according to the tree analysis described. A data set was divided into two nodes by tree analysis pattern, using one rule at a time in the form of a question. The presence or absence, and the intensity levels of one peak defined the splitting decision. This splitting process continued until the terminal nodes or leaves were produced or further splitting had no gain. Classification of terminal nodes was determined by the group ("class") of samples (i.e., lung cancer, healthy, or inflammatory) representing the majority of samples in that node. Peaks selected by this process formed the splitting rules to achieve the maximum reduction of cost in the two descendant nodes.

### Serum SAA determination

Serum levels of SAA were measured in human serum samples using a commercial enzyme-linked immuno-sorbent assay (ELISA) kit (Invitrogen, Camarillo, CA, USA), according to the manufacturers' instructions. ELISA tests were performed in triplicate on serum specimens from each patient in the study group. SAA levels were assayed in serum diluted by a factor of 200. If the absorbance readings exceeded the linear range of the standard curves, the ELISA assay was repeated after serial dilution of the supernatants.

### Statistical analysis

The SPSS software version 13.0 (SPSS INC.,Chicago, IL, USA) or Ciphergen ProteinChip Software, version 3.0.2 was used for statistical analysis and results were expressed as mean ± SD. Statistical significance was analyzed by using a two-tailed Student's t test and *p *< 0.05 was considered statistically significant. The discriminatory power for each putative marker was shown using the receiver operating characteristics (ROC) area under the curve (AUC).

## Results

### Reproducibility

**Figure 1:**
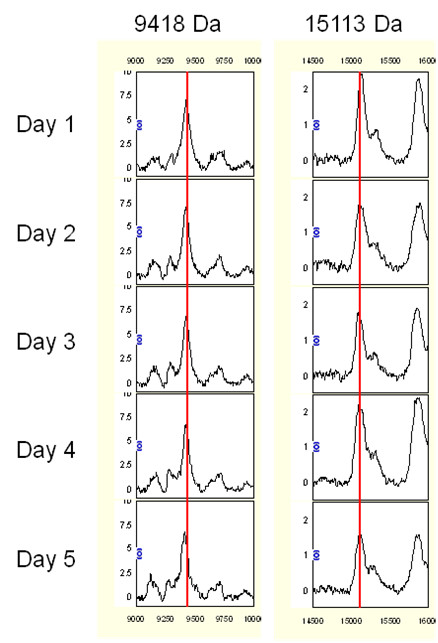
**Day-to-day verification of SELDI peaks at 9418 and 15113Da in the pooled normal sera**. The representative peaks from Days 1 to 5 are showed. Red lines, peaks used for calibration

To examine the reproducibility of the assay system, pooled normal sera from 60 individuals were used as control samples. Ten protein peaks randomly selected over the course of the study were used to calculate the coefficient of variance (CV) as described previously [33]. The reproducibility of the SELDI spectra was determined, both within and between arrays (intra-assay and inter-assay, respectively). The inter-assay (chip to chip) CV was 17.9% (14.9% to 20.3%) for peak intensity and 0.08% (0.056% to 0.102%) for mass accuracy (peak range: 5902-17340). The minimal variation of day-to-day instrumentation was shown in Figure 1.

### Detection of lung cancers by one stage SELDI profile

Serum samples were first analyzed from the training group using 134 random samples with or without lung cancer, using the SELDI Protein-Chip system. Peaks were detected automatically after baseline subtraction using Ciphergen ProteinChip Software, version 3.0.2. This analysis identified 51 signal peak protein clusters, seen in the spectrum representations of the two groups (cancer and non-cancer) within the 2,000 to 20,000 m/z range. No single peak could adequately discriminate lung cancer sera from healthy sera and inflammatory disease patients.

**Figure 2:**
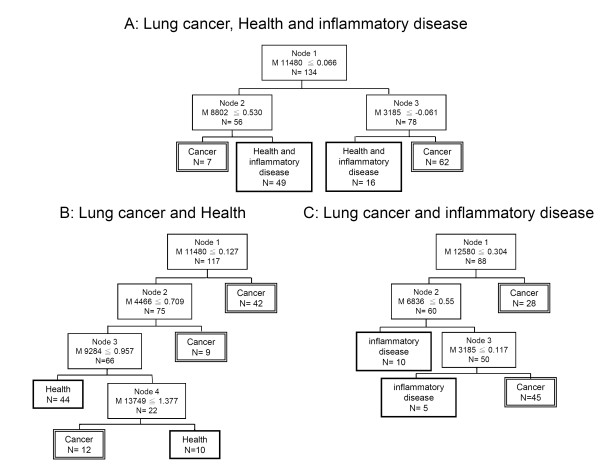
**Decision tree algorithms from three different training protocols**. **(A) **Serum samples of individuals with lung cancer and inflammatory disease, and healthy controls. **(B) **Serum samples of individuals with lung cancer and healthy controls. **(C) **Serum samples of individuals with lung cancer and inflammatory disease.

Using the spectrum, a decision tree classification algorithm was built and three protein peaks at 11480, 8802 and 3185 Da were automatically selected as splitters (*p *< 0.05, for each). The 11480 Da peak was used as the root node in the classification tree to divide the 134 samples into two groups (Figure 2A). The left node (node 2) included cases with peak intensity ≦ 0.53. The right node (node 3) contained the remaining with peak intensity ≦-0.061. Finally, all 134 cases in the training set were classified in the 4 terminal nodes, and a classification tree was obtained (Figure 2A). The sensitivity and specificity of the training set were 81.7% (58 of 71) and 85.7% (54 of 63), respectively. When the validity of this classification tree algorithm was challenged by the test set (87 cases), there was still high sensitivity (87.2%, 41 of 47) but low specificity (37.5%, 15 of 40) and accuracy (64.4%, 56 of 87) (Table 2).

**Table 2:** Performance characteristics of SELDI profiles in the diagnosis of lung cancer

**Training population**	**Training set**			**Test set**		
	**Sensitivity (%)**	**Specificity (%)**	**Accuracy (%)**	**Sensitivity (%)**	**Specificity (%)**	**Accuracy (%)**
Single-stage decision tree						
(A) Lung cancer + Healthy control & Inflammatory disease	81.7 (58/71)	85.7 (54/63)	83.6 (112/134)	87.2 (41/47)	37.5* (15/40)	64.4 (56/87)
(B) Lung cancer + Health control	88.7 (63/71)	89.1 (41/46)	88.9 (104/117)	83.0 (39/47)	84.6 (22/26)	83.6 (61/73)
(C) Lung cancer + Inflammatory disease	94.4 (67/71)	70.6 (12/17)	89.8 (79/88)	85.1 (40/47)	57.1 (8/14)	78.7 (48/61)
Two-stage decision tree						
	80.3 (57/71)	88.8 (56/63)	84.3 (113/134)	70.1 (33/47)	80.0* (32/40)	74.7 (65/87)

*p < 0.05 comparing single-stage vs. two-stage decision tree patterns in specificity

The training set was further divide into two cohorts. The first cohort was lung cancer patients and healthy control. The decision tree was shown in Figure 2B. The 11480 Da peak was used as the root node in the classification tree and followed three peaks to classify the cancer and healthy cohorts. The sensitivity and specificity of the training set were 88.7% (63 of 71) and 89.1% (41 of 46), respectively, and 83% (39 of 47) and 84.6% (22 of 26), respectively, as validated in the test group. The second cohort was lung cancer and inflammation patients. The decision tree was classified by three peaks as shown in Figure 2C. The 12580 Da peak was used as the root node in this classification tree. The sensitivity and specificity from the training set were 94.4% (67 of 71) and 70.6% (12 of 17), respectively, and 85.1% (40 of 47) and 57.1% (8 of 14), respectively, in the test set.

### Detection of lung cancers by two stage SELDI profile

**Figure 3:**
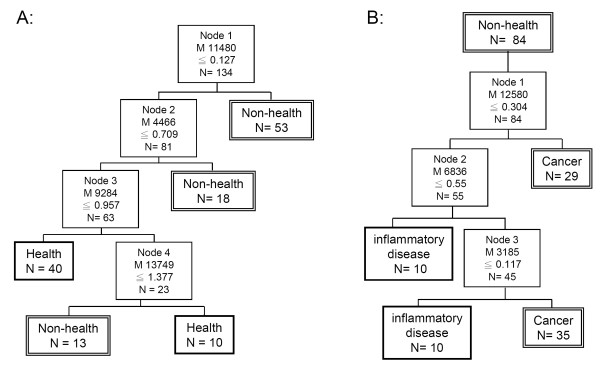
**Decision tree algorithm of the two-stage training protocol**. **(A) **Using the decision tree algorithm B in the one-stage training protocol for different health and non-health groups. **(B) **Putting the non-health group into the decision tree algorithm **C **in the one stage training protocol to differentiate lung cancer and inflammatory disease.

To further improve the outcome of lung cancer detection rate, the two original cohorts shown in Figures 2B and C were re-combined into a two-stage training set as shown in Figure 3. The validity of this classification tree algorithm was then challenged by the test set. The first stage aimed to distinguish the healthy controls from cancer patients and patients with inflammation using four peaks. Any non-healthy control was subjected to further analysis using three peaks led by the 12580 Da node to distinguished cancer versus non-cancer inflammatory disease. The two-stage training set had a sensitivity of 80.3% (57/71) and specificity of 88.8% (56/63) in the training set, which was 70.1% (33/47) and 80% (32/40), respectively, in the test group (Table 2). The accuracy in the test group was 74.7% (65/87).

**Figure 4:**
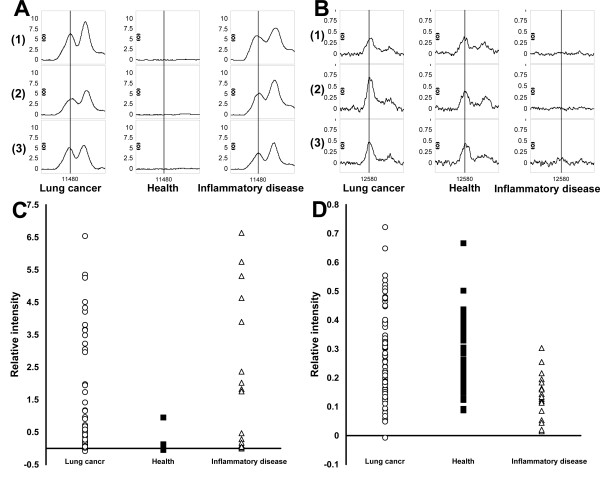
**The splitting peaks in two-stage training protocol and the density peaks of these two proteins in all sera samples**. **(A) **The first splitting peak from the first-stage training set marker showing a protein peak marker of 11480 Da on the CM10 chip (vertical line). Protein mass spectra were shown in sera of individuals with lung cancer (left), healthy control (middle), and inflammatory disease (right). **(B) **The first splitting peak of the second-stage training set marker showing a protein peak of 12580 Da (vertical line). **(C) **Density peak of 11480 Da protein in the sera of all samples. **(D) **Density peak of 12580 Da protein in the sera of all samples.

Comparing the one-stage method to the two-stage method in the testing group, the two-stage method had higher specificity and accuracy (*p *< 0.05). The represented peaks from the two-stage training set were shown in Figures 4A and 4B. In first-stage training set, the peak was 11480 Da protein. This peak was increased in both lung cancer and inflammatory disease patients, but was very low in the health controls (Figure 4C). In the second-stage training set, the protein peaks with 12580, 6836, and 3185 Da was used to separate cancer patients from non-cancer inflammatory disease. The average peak intensity of 12580 was higher in lung cancer patients and health controls, but relatively lower in inflammatory disease patients (Figure 4D). The predominately expressed protein peak at 11480 Da was the primary splitter (distinguishing factor) in all classification trees generated to separate healthy individuals, which were most likely to have SAA with average m/z countered around this area [22,25,26,34].

**Figure 5:**
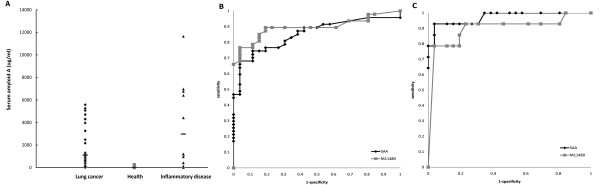
**SAA level in the sera of test group and the ROC curve of SAA and 11480 Da peak intensity**. **(A) **The protein level of SAA determined by ELISA from the test group, including those with lung cancer and inflammatory disease, and healthy controls. Horizontal bar, mean values. **(B) **The ROC curve generated by comparing healthy control and lung cancer patients with SAA and 11480 Da peak intensity. **(C) **The ROC curve generated by comparing healthy control and those with inflammatory disease, by SAA and 11480 Da peak intensity.

The SAA ELISA was used to confirm the value of SAA as first splitter. The SAA was elevated in most sera of lung cancer and inflammatory disease as compared to healthy control (*p *< 0.02), but not significantly different between lung cancer and inflammatory disease (Figure 5A). The Receiver Operating Characteristics (ROC) curve for SAA compared healthy control versus lung cancer or inflammatory disease (Figures 5B,5C). The areas under the curve were 0.822 for SAA vs. 0.864 for 11480 Da (Figure 5B) and 0.946 for SAA vs. 0.874 for 11480 Da (Figure 5C). This indicated that SAA was a good marker to discriminate inflammatory disease and lung cancer from healthy controls, but not sensitive enough to differentiate between lung cancer and inflammatory disease.

## Discussion

This study has shown that inflammatory disease can severely interfere with the specificity of detecting lung cancer by one-stage training SELDI profiles. Using a two-stage training process can effectively improve the discrimination of lung cancer from inflammatory disease. The purer and simpler clinical sample classification in the test group can achieve more reliability in the final decision tree made by SELDI. Although it seems to be a simple idea, it has not been clearly validated in previous SELDI literature.

Currently, there are no satisfactory serum biomarkers suitable for screening and early diagnosis of lung cancer. SELDI is a high through-put technique used to generate protein expression profiles which, in combination with bioinformatics tools to extract information for biomarker discovery, has been essential in identifying novel protein biomarkers. The SELDI-TOF-MS analysis is divided into a pattern discovery and a pattern-matching phase. This technology has shown great potential for the early detection of ovarian, breast, prostate, liver and lung cancers from healthy individuals [9-13]. It is generally believed that the training samples should include randomly selected subjects without cancer and subjects with cancer for analysis.

Nonetheless, most of the studies focus on distinguishing cancer patients from healthy controls. Because inflammatory diseases are commonly associated with cancer patients and malignancies are often connected to inflammatory response for the host, the inclusion of benign disease connected to an inflammatory response is crucial in obtaining a cancer profile. This study investigates not only the influence of inflammatory disease in the differential diagnosis of lung cancer but also point out that a two-stage decision process is more accurate than a single-step process.

In the current study, peaks at 11480, 4466, 9284, 13749 kDa are over-expressed in the lung cancer group, which can be distinguished from healthy individuals and is compatible with previous studies [15,19,25]. In previous studies, Han et al. [19] used SELDI-TOF protein analysis to distinguish lung cancer patients from healthy individuals and demonstrated high sensitivity (89%, 81 of 89) and specificity (91%, 86 of 96). That study suggested the use of tumor markers such as CEA. However, inflammatory disease will interfere with the detection of lung cancer by SELDI analysis if this method is applied clinically. The current study is therefore designed to evaluate the influence of inflammatory disease and use a novel two-stage method to improve the detection rate.

Yang et al [15] used SELDI proteomic pattern to distinguish lung cancer and healthy individuals using a one-stage decision tree. They demonstrated good sensitivity (86.9%, 73 of 84) and specificity (80%. 24 of 30). But how the inflammatory status contaminated their lung cancer samples can't be evaluated in their study. According to our results, subjects not belonging to "pure healthy" population will have to go through another 3 protein discrimination at 12580, 6836, and 3185 kDa obtained from the secondary stage training with the population of lung cancer and inflammatory disease to further distinguished lung cancer. Follow-up peaks identification and validations are needed.

The peak of 11480 Da is suspected to be SAA from similar m/z size. The m/z of SAA ranges from 11400 to 11800 Da in different studies [34]. SAA is a non-specific inflammatory marker found in inflammatory diseases and is elevated in many malignancies, such as ovarian, prostate, renal, and lung cancer [24-26]. Both SAA levels and peak 11480 intensity levels have similar AUC from ROC curve analysis from the samples here, which may serve as indirect evidence that SAA mimics protein peak 11480 Da [14,21-27]. This study aimed to increase the detection accuracy by partitioning analysis into two stages. We found that protein peak 11480 Da was the primary splitter regardless one- or two-stage analysis. It should be important to perform protein identification about this peak because proteins other than SAA may also give a peak of this m/z. SAA is a non-specific acute phase protein induced by host inflammatory response to stimuli from liver. Recently, SAA is found to be highly expressed in lung cancer tissue and it can be induced when lung cancer cells are cocultured with macrophage or cytokines [35]. These properties may place SAA an important role for tumor pathogenesis within tumor microenvironment[36]. Elevated SAA may be a primary product of tumor cells as well as hepatocytes. We believed SAA can be served as clinical biomarkers for lung cancer prognosis and treatment guidance but not diagnosis. This opinion warrants further investigation.

Screening biomarker patterns for diagnosis of lung cancer using SELDI-TOF has been hindered by the false-positive results due to inflammatory disease. These concerns become significant when clinically applied for cancer screening. Our study provided a simple idea of using two-stage method to lower the influence of inflammatory disease. Although it seems like a simple idea, it has not been clearly validated in previous SELDI literature. So we hope the manuscript may contribute something in this regard. Reproducibility in different laboratories by different investigators, even with the same chips, will remain difficult to standardize and validate. There is still a long way to go before the SELDI platform can be used as a clinical diagnostic tool.

## Conclusions

This study reveals the influence of inflammatory disease in the detection of lung cancer using the SELDI profile, which needs to be considered given the increasing use of proteomics for cancer diagnosis. It is possible to examine mass spectra derived from a two-stage decision tree as shown here. The two-stage test sample stratification based on inflammatory status will improve the false positive rate and be more useful in differentiating lung cancer patients from healthy and non-cancer inflammatory disease populations.

## Competing interests

The authors declare that they have no competing interests.

## Authors' contributions

PSH wrote the main manuscript and performed the data analysis. YSW and SCH performed the experiments. YHL contributed to the statistical analysis. CCC, YHT, JSJ and SJK collected lung cancer patients serum samples and explain the inform of consent to patients. WCU and KHC participated in the design of the experiments, supervised the data analysis and interpretation, and participated in manuscript writing. All authors read and approved the final manuscript.
